# The goal of ape pointing

**DOI:** 10.1371/journal.pone.0195182

**Published:** 2018-04-25

**Authors:** Marta Halina, Katja Liebal, Michael Tomasello

**Affiliations:** 1 Department of History and Philosophy of Science, University of Cambridge, Free School Lane, Cambridge, United Kingdom; 2 Department of Education and Psychology, Cluster Languages of Emotion, Evolutionary Psychology, Freie Universität Berlin, Berlin, Germany; 3 Department of Developmental and Comparative Psychology, Max Planck Institute for Evolutionary Anthropology, Leipzig, Germany; 4 Department of Psychology & Neuroscience, Duke University, Durham, North Carolina, United States of America; University of Portsmouth, UNITED KINGDOM

## Abstract

Captive great apes regularly use pointing gestures in their interactions with humans. However, the precise function of this gesture is unknown. One possibility is that apes use pointing primarily to direct attention (as in “please look at that”); another is that they point mainly as an action request (such as “can you give that to me?”). We investigated these two possibilities here by examining how the looking behavior of recipients affects pointing in chimpanzees (*Pan troglodytes*) and bonobos (*Pan paniscus*). Upon pointing to food, subjects were faced with a recipient who either looked at the indicated object (successful-look) or failed to look at the indicated object (failed-look). We predicted that, if apes point primarily to direct attention, subjects would spend more time pointing in the failed-look condition because the goal of their gesture had not been met. Alternatively, we expected that, if apes point primarily to request an object, subjects would not differ in their pointing behavior between the successful-look and failed-look conditions because these conditions differed only in the looking behavior of the recipient. We found that subjects did differ in their pointing behavior across the successful-look and failed-look conditions, but contrary to our prediction subjects spent more time pointing in the successful-look condition. These results suggest that apes are sensitive to the attentional states of gestural recipients, but their adjustments are aimed at multiple goals. We also found a greater number of individuals with a strong right-hand than left-hand preference for pointing.

## Introduction

Great apes use gestures regularly to communicate with conspecifics and with humans. Many of these gestures are employed in a flexible and goal-directed manner. Gestures are used flexibly in the sense that signalers use multiple gestures for a single communicative end and a single gesture for multiple communicative ends [1, 2]. Signalers also modify their gestural strategies depending on the attentional state and response of the recipient ([3–9]; see [10] for related findings in monkeys). Gestures are used in a goal-directed manner in that signalers wait for an appropriate response and persist in the face of failure [4, 7, 11].

In the last few years, considerable progress has been made in understanding the goals of ape gestures [12, 13]. Generally, apes use gestures in order to request that another agent perform a particular social action. These *action-soliciting gestures* include communicative attempts to get others to play, travel, groom, and nurse [11, 12, 14–16]. Apes also use *attention-getting gestures* (or “attention-getters”) in order to attract the attention of other agents [1, 17]. Gestures such as *poke*, *throw stuff*, and *hand-clap* attract the attention of other agents through their tactile and auditory effects, but apes use these gestures communicatively as evidenced by the fact that signalers make eye contact with the recipient and wait for a response [1, 18]. Chimpanzees appear to employ attention-getters with the specific aim of attracting attention, as they use them more when a recipient is not attending [3, 8, 17, 19, 20–22]. This sophisticated use of attention-getters is consistent with what is known about great ape visual perspective taking abilities. Great apes understand what agents can see given the orientation of their body, head, and eyes, and how perceptions are affected by objects such as transparent barriers, opaque barriers, and even mirrors [9, 23–27]. They take these perceptions into account when deciding how and when to gesture, how and when to compete over food, etc. [5, 8, 28–30].

Apes thus have the capacity to communicatively manipulate the attentional states of others. One unanswered question, however, is whether they also use these skills to direct the attention of others in a triadic manner to a third object, agent, or location. A strong candidate for such an *attention-directing gesture* is pointing. Apes regularly use pointing gestures in captivity [31, 32] and have occasionally been observed pointing in the wild [33–35]. Ape pointing is clearly a communicative act, rather than an action attempt, such as a failed reach: when pointing, apes typically extend their finger or hand toward an object, alternate their gaze between the object and the recipient of their gesture, wait for a response, and when the recipient acts on the indicated object, appear satisfied with the outcome [32, 36]. Apes also point only when another agent is present [36].

What is the aim of ape pointing gestures? We know that chimpanzees point referentially or to indicate a particular object, as they persist pointing at a food item even when they receive another (non-indicated) food item [32, 37]. Additional studies suggest that captive apes do not use pointing with the sole aim of requesting an object because they also point to objects (such as a hidden tool) that they themselves do not want, but that a human needs to fulfill a task (such as obtaining food that is desired by the signaler) [38–40]. One possibility concerning the goal of ape pointing is that apes point with the primary aim of directing the attention of another agent to a particular object—that is, that they use this gesture analogously to attention-getters, but with the aim of directing attention triadically to objects other than the self. This hypothesis is consistent with what is known about ape pointing thus far: apes might point at an object knowing that once a recipient *sees* that object, he or she will likely perform some further desired action on it (such as hand it over, if it’s food, or use it, if it’s a tool). This hypothesis is also supported by independent observations of triadic gestures in great apes. Savage-Rumbaugh and McDonald [41], for example, described the human-enculturated bonobo Kanzi as presenting objects (such as forbidden mushrooms) to the gaze of human caretakers (see also the “directed-scratch” gesture in [42, 43]: “ball offer” in [44]; and “food offer” in [15]). In cases such as these, it is possible that apes are using gestures in order to bring an object or body part to the attention of another agent [45]. The question with respect to pointing is whether it serves a similar communicative function: that is, whether apes point with the goal of directing a recipient’s attention to the indicated object.

We address this question here by applying the gestural-persistence research paradigm to pointing in an experimental setting [7, 12, 13, 32, 46]. In this paradigm, one hypothesizes the goal of a gesture (the “presumed goal” in the vocabulary of [12]). This presumed goal is then tested against a signaler’s persistence given different outcomes. When a signaler ceases gesturing, this suggests that the goal of the gesture has been achieved (goal-outcome match). If instead a signaler persists gesturing, this suggests that the goal of the gesture has not been achieved (goal-outcome mismatch). In order to assess the goal of ape pointing, we presented chimpanzees and bonobos with a situation that would lead them to spontaneously produce pointing gestures for a human experimenter (E). In this situation, food was visible (but not accessible) to the apes and not visible (but accessible) to E. We then hypothesized two goals for ape pointing in this context: to 1) direct attention or 2) request food. These hypothesized goals were then tested experimentally. In one experimental condition, E responded to the pointing gestures produced by subjects by looking at the indicated object (“successful look”); in a second condition, E responded to pointing gestures by looking at a location other than that indicated by the subject (“failed look”). We predicted that if directing attention is the primary goal of ape pointing, then subjects should persist pointing in the failed-look condition (due to the goal-outcome mismatch) and cease or decrease the time spent pointing in the successful-look condition (due to the goal-outcome match). It is worth noting that the latter prediction does not entail that subjects cease communicating with E altogether. Instead, once subjects have succeeded in directing E’s attention to the food, they might employ alternative gestures aimed at requesting food, such as begging gestures [1, 14]. Language-trained chimpanzees have been shown to produce higher rates of non-indicative gestures like head bobbing (and lower rates of indicative gestures like pointing) when an experimenter who is searching for hidden food points successfully towards the food rather than elsewhere [47]. We anticipated something similar in response to the (correct versus incorrect) looking behavior of the experimenter here. Apart from spending less time pointing, we made no predictions about what subjects would do once they succeeded in directing E’s attention. Finally, we expected that if apes point in this context with the primary goal of requesting food, then their pointing behavior should not differ across the successful-look and failed-look conditions because these conditions only differ in E’s looking behavior.

In addition to the above, we examined the hand preferences of subjects while pointing. Handedness of manual gestures in apes is thought to be an evolutionary precursor to the left hemisphere lateralization of language and speech production in humans [48]. Previous studies have found that apes exhibit a right-hand preference when using manual gestures accompanied by vocalizations, suggesting that some great ape communicative behaviors are also lateralized to the left hemisphere [49]. We examined whether subjects in this study exhibited a similar right-hand preference when pointing.

## Methods

### Subjects

Five bonobos (two females and three males) and eighteen chimpanzees (twelve females and six males) participated in this experiment. All of the subjects were born in captivity and housed at the Wolfgang Köhler Primate Research Center (WKPRC) in Leipzig, Germany. Subjects ranged in age from 2 to 34 years. Two of the bonobos were mother reared, while three were human reared. Out of the chimpanzees, twelve were mother reared and six were human reared. Throughout the study, the subjects remained on their normal dietary routine and had unlimited access to water. They also had daily access to a wide variety of enrichment activities and objects, including trees, sticks, ropes, balls, shaking boxes, poking bins, jute and paper parcels, bamboo stick treats, and more (see http://wkprc.eva.mpg.de/english/files/enrichment.htm).

Rearing conditions are known to affect the social and communicative abilities of human and nonhuman primates, including their ability to engage in triadic interactions [50]. Although we did not examine the effects of rearing history here, it is worth highlighting the conditions under which the five human-reared subjects in the main test of this study (see below) were raised. Annet and Alexandra were both cared for by humans from birth, having been rejected by their biological mothers. They spent the first two years of their lives at a medical research facility and moved to the Wolfgang Köhler Primate Research Center (WKPRC) at around 22 months of age. During their time at the medical facility and for their first year at the WKPRC, their contact with humans consisted mostly of care-giving activities. For approximately 2 years after that, they had more extensive interactions with humans and human artifacts, although these interactions were not systematic [51]. Alex was raised in a human home until approximately one year of age. During this time, he had continuous interaction with humans, being treated similarly to a human child. At around 14 months, he came to the WKPRC and continued to interact with humans and human artifacts for approximately 6 hours per day [51]. This continued until he was approximately 45 months old. The bonobo Limbuko was cared for from birth by humans at a medical facility nursery in Stuttgart. The practices at the facility at the time were that individuals who lost contact with their mothers should receive more motherly care from their human caretakers. Thus Limbuko had more interaction with humans than Alexandra and Annet during these early years. He received no additional human rearing after arriving at the WKPRC at five years of age. Unfortunately, little is known about the chimpanzee Natascha’s history, except that she came from a biomedical facility and had close contact with humans before she arrived in Leipzig at 20 years old.

This research was conducted at the WKPRC and strictly adhered to the legal and institutional requirements of that location. All procedures were non-invasive and research complied with the recommendations of the Weatherall report, the EAZA Code of Practice Article 4: Research, and the WAZA Ethical Guidelines for the Conduct of Research on Animals by Zoos and Aquariums. This research was also ethically approved by an internal ethics committee at the Max Planck Institute for Evolutionary Anthropology consisting of scientists (Prof. M. Tomasello, Dr. J. Call, Dr. D. Hanus), zoo keepers (head keeper F. Schellhardt, assistant head keeper M. Lohse), and a veterinarian (Dr. A. Bernhard).

### General setup

**Fig 1:**
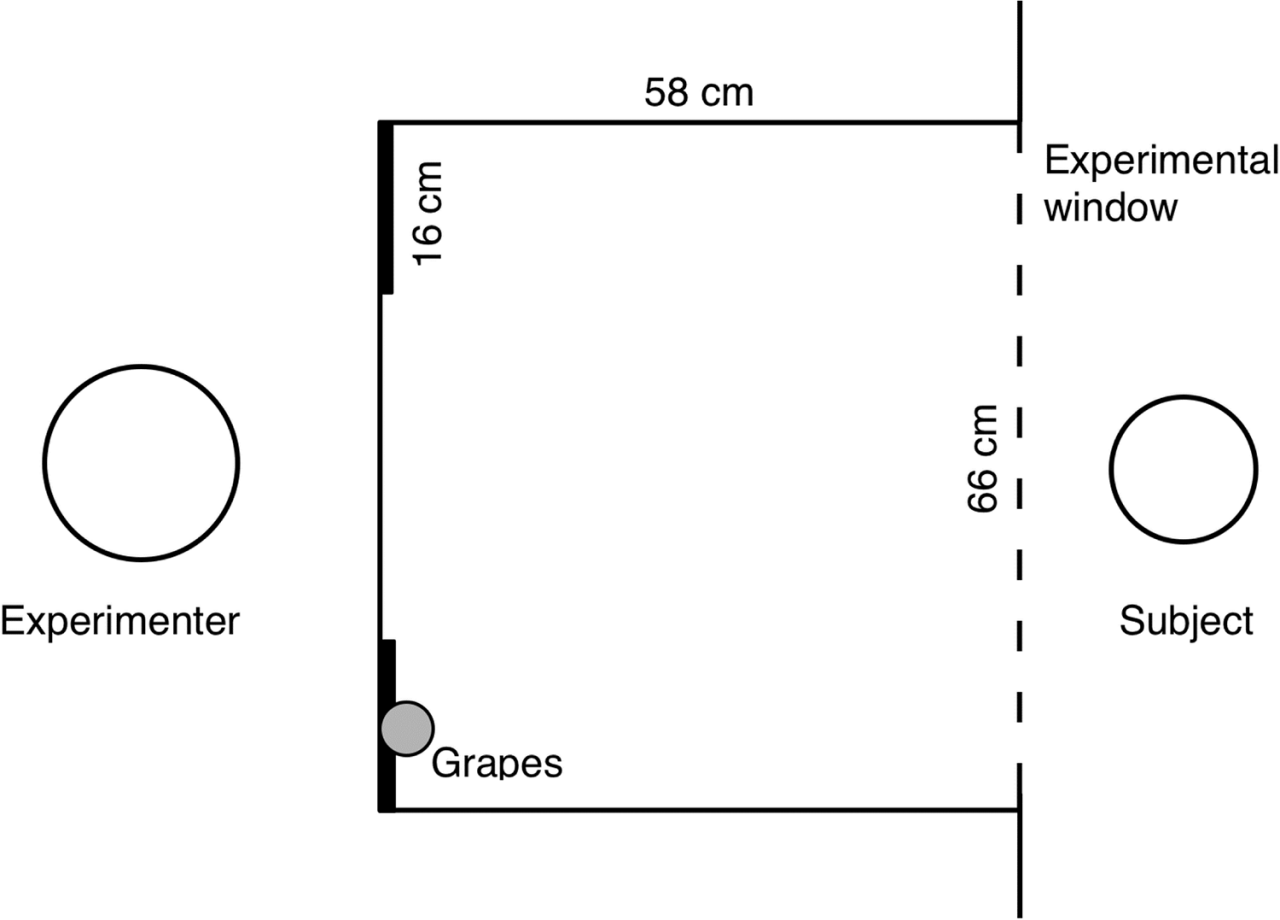
The general experimental setup for all conditions.

Subjects were tested individually in a testing room. Those younger than five years of age could move between the testing room and an adjacent room occupied by their mother. The experimenter sat in an area adjoining the testing room and interacted with the subject through a wire-mesh experimental window that was 66 cm wide and 48 cm high. Attached to the bottom edge of the experimenter’s side of the window was a 58 × 66 cm table where the experimenter sat directly across from the experimental window and about 90 cm away from the window (Fig 1). For safety reasons, two clear Plexiglas walls and a clear Plexiglas ceiling enclosed the table. When giving a grape to the subject, the experimenter gently rolled it across the table to the experimental window, where the subject could pick it up through the mesh panel.

Two small (16 cm wide × 21 cm high) occluders were positioned between the experimenter and the experimental window—one to the right of the experimenter and one to the left. The occluders were located approximately 58 cm from the experimental window with their bottom edges 27 cm above the table and their inner edges 34 cm apart. Five grapes (attached to a grapevine) hung on the subject’s side of one of the small occluders (with the location of the grapes counterbalanced across trials). In this way, the grapes were fully visible to the subject from the vantage point of the experimental window, but could not be seen by the experimenter unless she leaned forward and turned her head to the side. A large occluder was positioned between the experimental window and the two small occluders, blocking the experimental setup from the subject until a trial began (see main test below).

Two video cameras were located on each side of the experimenter and recorded the behavior of the subject at the experimental window in all conditions. A third video camera recorded the experimenter during the successful-look and failed-look conditions. All coding was based on the footage from these cameras (see coding procedures below).

### Pretest

The purpose of the pretest was to identify those individuals that would use pointing gestures in order to indicate or request the grapes in the above experimental setup. All subjects participated in this pretest, which consisted of twelve two-minute trials. In order to pass the pretest, a subject had to direct a pointing gesture at the grapes while making eye contact with the experimenter at least once during eight of the twelve trials. Three bonobos and ten chimpanzees met this requirement. Four of the subjects that participated (and passed) this pretest had previously participated in an experiment with a similar setup. However, this previous experiment involved no training. During the pretest, the experimenter responded to the subject as in the motivational condition described below.

### Main test

Three bonobos and nine chimpanzees participated in the main test, all of which had passed the pretest (Table 1). One subject who passed the pretest did not participate in the main test for reasons unrelated to the study. Before a trial began, the experimenter sat at the table across from the experimental window. In the case of subjects younger than five years, the experimenter waited until the subject entered the testing room and called the subject, if necessary. Subjects had to be in the testing room for a trial to begin, but did not need to be positioned in front of the experimental window. A trial began when the experimenter removed the large occluder positioned between the experimental window and the experimental setup, and ended with the experimenter replacing the large occluder. Each trial lasted two minutes.

**Table 1:** Main test participant information.

Species	Name	Sex	Age (year.month)	Reared by
Chimpanzee	Kofi	Male	5.1	Mother
	Kara	Female	5.2	Mother
	Alex	Male	9.6	Human
	Pia	Female	10.11	Mother
	Alexandra	Female	11.0	Human
	Annet	Female	11.0	Human
	Sandra	Female	17.2	Mother
	Jahaga	Female	17.8	Mother
	Natascha	Female	30.5	Human
Bonobo	Fimi	Female	2.1	Mother
	Luiza	Female	5.7	Mother
	Limbuko	Male	14.10	Human

The main test consisted of three conditions: one motivational and two experimental (successful-look and failed-look). In all three conditions, the experimenter acted in the following ways: First, she responded only to pointing gestures (as defined in the section “Identifying pointing gestures in real time” below); otherwise, she sat silently and causally watched the subject throughout the duration of the trial. Second, if the subject pointed to any location not occupied by the grapes, the experimenter followed the pointing gesture to the indicated location and alternated her gaze between the subject and that location. Third, if the subject pointed to the experimenter, the experimenter lifted her hands to show that she held nothing. The experimenter’s behavior only differed across the three conditions in response to the situation in which the subject directed a pointing gesture at the grapes. In this case, the experimenter responded in one of three ways:

1. Motivational condition. The experimenter alternated her gaze between the subject and the grapes, picked one grape, and gave it to the subject.
2. Successful-look condition. The experimenter alternated her gaze between the subject and the grapes, but did not pick a grape and give it to the subject.
3. Failed-look condition. The experimenter alternated her gaze between the subject and a location not indicated by the subject (namely, the subject’s side of the second small occluder rather than the occluder with the grapes) and did not pick a grape and give it to the subject.

The experimenter performed one of these three actions in response to all pointing gestures directed at the grapes. If a subject pointed at the grapevine in the motivational condition when no grapes remained, the experimenter gave the grapeless grapevine to the subject.

All subjects participated in a total of two successful-look sessions, two failed-look sessions, and four motivational sessions, where a session consisted of two successive trials (Table 2). The order of conditions was counterbalanced across subjects. One experimenter ran all of the motivational trials, while two experimenters ran the successful-look and failed-look conditions (with the experimenter assigned to each experimental condition counterbalanced across subjects). Subjects received the three different conditions from three different experimenters, but had the same experimenter for all trials within a condition (Table 2). All subjects received one grape between trials (which was brought in from outside of the testing room) regardless of their performance during a trial.

**Table 2:** Main test design.

Day	Experimenter	Condition	Session	Number of trials
1	A	Motivational	1	2
	B	Failed-look	1	2
2	A	Motivational	2	2
	C	Successful-look	1	2
3	A	Motivational	3	2
	B	Failed-look	2	2
4	A	Motivational	4	2
	C	Successful-look	2	2

### Identifying pointing gestures in real time

In order to run this experiment, the experimenters had to identify pointing gestures in real time. For this purpose, following the literature, we defined a *pointing gesture* as the mechanically ineffective act of extending a finger, hand, and/or arm through the wire-mesh experimental window and directing it at some point beyond this window, while making eye contact with the experimenter at least once during the process of extending, maintaining, or retracting the extended part [1, 52].

Gestures that resembled pointing but included an upward-facing palm only counted as pointing if they were not directed at the experimenter; if they were directed at the experimenter, they were excluded as begging gestures. The extension of a finger or hand in close proximity to the table (that is, ≤ 8 cm above the table) also did not count as a pointing gesture, as this was the area through which subjects retrieved grapes, making it difficult to rule out the possibility of an attempted mechanically effective action (attempting to retrieve a grape).

### Coding procedures

We coded the behavior of subjects by analyzing video taken during the experiment. Coders were blind to which condition was in effect during coding. We recorded all pointing gestures and coded their duration (in seconds) in order to determine if subjects spent more time pointing per trial during the failed-look condition compared to the successful-look condition. We defined a pointing gesture as beginning when the subject’s finger, hand, or arm reached its final extended position (that is, the last position before retraction) and ending when the subject began retracting and/or changing the orientation of the extended part (for alternative ways of parsing gestural units based on human studies, see [53–55]). It is important to note that we measured pointing durations in order to determine the total time that subjects spent pointing per trial rather than the average duration of a single point.

Following previous studies, we relied on gestural persistence as a measure of signaler dissatisfaction. However, rather than measuring persistence in terms of pointing frequency, we measured it in terms of the total time spent pointing per trial. We did this because we found that the durations of individual points varied widely (from <1 to 25 seconds) for reasons that seemed unrelated to the question under investigation. The presence of a mesh panel between the subject and the grapes meant that if a subject wanted to adjust the direction of her point, she had to remove her arm or finger from one hole in the mesh and insert it into another. For coding purposes, this counted as a new point. However, such adjustments in this context did not necessarily reflect a subject’s motivation to point. Thus, we reasoned that it was the total time spent pointing per trial that mattered here, rather than the frequency. Our reliance on time spent pointing represents a divergence from pervious studies on gestural persistence [36]. In order to enable the comparison of these two measures, we also provide data on gestural frequencies here (see Results and S1 Table).

In addition to recording those pointing gestures produced while making eye contact with the experimenter, we recorded those behaviors that met our criteria for a pointing gesture except that the subject did not make eye contact with the experimenter. We called these latter events “points without eye contact” (as opposed to “points with eye contact”). A widely accepted criterion for intentional gestural communication is that the signaler make eye contact with the recipient of the gesture [1, 11, 56, 57]. Thus, we compared the time spent pointing with and without eye contact in order to confirm that the types of behaviors that we were identifying as pointing gestures were in fact generally used in a communicative manner, as indicated by the signaler establishing eye contact with the experimenter.

For the successful-look and failed-look conditions, we also recorded the times at which the experimenter alternated his gaze during a trial—an action that was performed only in response to points with eye contact. The purpose of this was to confirm that the points with eye contact identified through video analysis matched those identified by the experimenter in real time. We confirmed this by comparing the times that points with eye contact occurred with the times at which the experimenter alternated his gaze. If the experimenter alternated his gaze during a point that was coded as “without eye contact” or ≤ 1 second after the occurrence of a point coded as “without eye contact,” then we counted it as a point with eye contact, as this is what the experimenter identified in real time.

We classified all pointing gestures according to whether they were directed at the grapes or directed at a location other than that occupied by the grapes (where the possible pointing directions included left, center, and right; and the location of the grapes varied from left to right—see General setup). We did this in order to determine whether subjects directed most of their pointing gestures at the grapes, as would be expected if they were using this gesture in a referential manner. Referential gestures are communicative acts that indicate a specific entity [36]. They require both engagement with a recipient (e.g., in the form of eye contact) and being directed at an object (e.g., by orienting the gesturing part, such as a finger or hand, toward an object). We identified referential gestures here as those points with eye contact that were oriented toward the grapes. We also recorded how long a subject spent away from the experimental window during a trial in order to assess the degree of subject participation in the experiment over the course of the study. The first author coded the pointing gestures produced by the subjects (including their duration, orientation, and the presence or absence of eye contact) and the time at which the experimenters alternated their gaze. An assistant coded the time that subjects spent away from the experimental window. Inter-observer reliability was assessed in the following ways: for time spent away from the experimental window, the first author independently coded 20% of the successful- and failed-look trials. Agreement was high (Spearman correlation: *r*_*s*_ = 0.988, *N* = 38, *p* < 0.001) and there was no difference in the durations identified by the two coders (Wilcoxon signed ranks test: *p* = 0.457). For the direction and duration of pointing gestures, an assistant independently coded 10% of the 1,680 pointing gestures produced. Agreement was good on the orientation (left, right, center) of points (Cohen’s Kappa = 0.741) and the duration of points (Spearman correlation: *r*_*s*_ = 0.858, *N* = 168, *p* < 0.001) with no difference between the two coders in their assessment of pointing durations (Wilcoxon signed ranks test: *p* = 0.733). For experimenter gaze-alternation times, an assistant independently coded 13 out of the 96 experimental trials. The assistant identified 125 instances of experimenter gaze alternation occurring during these trials, while the original coder independently identified 126. There was excellent agreement on the precise time (in seconds) that the gaze alternations occurred (Spearman correlation: *r*_*s*_ = 0.999, *N* = 126, *p* < 0.001.) with no difference between the times identified by the two coders (Wilcoxon signed ranks test: *p* = 0.763). For the presence and absence of eye contact made by subjects while pointing, an assistant independently coded 10% of the 1,680 pointing gestures produced. Eye contact agreement was fair (p_o_ = 85%, Cohen’s kappa = 0.554).

All analyses were done using nonparametric tests (Friedman tests for several comparisons and Wilcoxon tests for pairwise comparisons). Due to small sample sizes, we did not analyze species differences.

## Results

### Intentionality and hand preference

If subjects used their pointing gestures in a communicative manner, then they should have spent more time employing points with eye contact than without eye contact, regardless of the condition. We found that this was the case (Wilcoxon signed ranks tests: motivational condition *Z* = -2.98, *p* = 0.001, successful-look condition *Z* = -3.06, *p* < 0.001, failed-look condition *Z* = -3.06, *p* < 0.001). For the motivational, successful-look, and failed-look conditions, subjects spent an average of 27, 25, and 18 seconds respectively pointing with eye contact per trial. In contrast, they spent an average of 2 to 3 seconds pointing without eye contact per trial. Given that the inter-observer reliability for presence and absence of eye contact was moderate, however, our central analysis includes both points with eye contact and all points (with and without eye contact pooled together).

Out of the 1,190 points with eye contact, 66.8% were produced with the right hand. We used the handedness index (HI) to determine the hand preference of individuals when pointing with eye contact [58–60]. HI is equal to the number of right-handed events minus the number of left-handed events divided by the total number of events or (R–L)/(R + L). The calculated value ranges from -1.0 to 1.0 with the sign indicating the hand preference (negative indicating left and positive indicating right) and the absolute value indicating the strength of the hand preference. We found that individuals varied in their hand preferences with four subjects exhibiting a strong right hand preference (HI > 0.80) and one exhibiting a strong left hand preference (HI = -1.0) (Table 3).

**Table 3:** Handedness of pointing subjects.

Name	Number of points produced*	Handedness Index (HI)
Alex	94	-1.00
Alexandra	93	-0.57
Jahaga	141	-0.46
Limbuko	80	-0.25
Fimi	155	-0.15
Luiza	174	-0.14
Pia	99	0.31
Annet	129	0.57
Sandra	131	0.89
Kofi	323	0.98
Kara	308	1.00
Natascha	65	1.00

*Points with eye contact

### Did subjects spend more time pointing per trial in the failed-look than in the successful-look condition?

**Fig 2:**
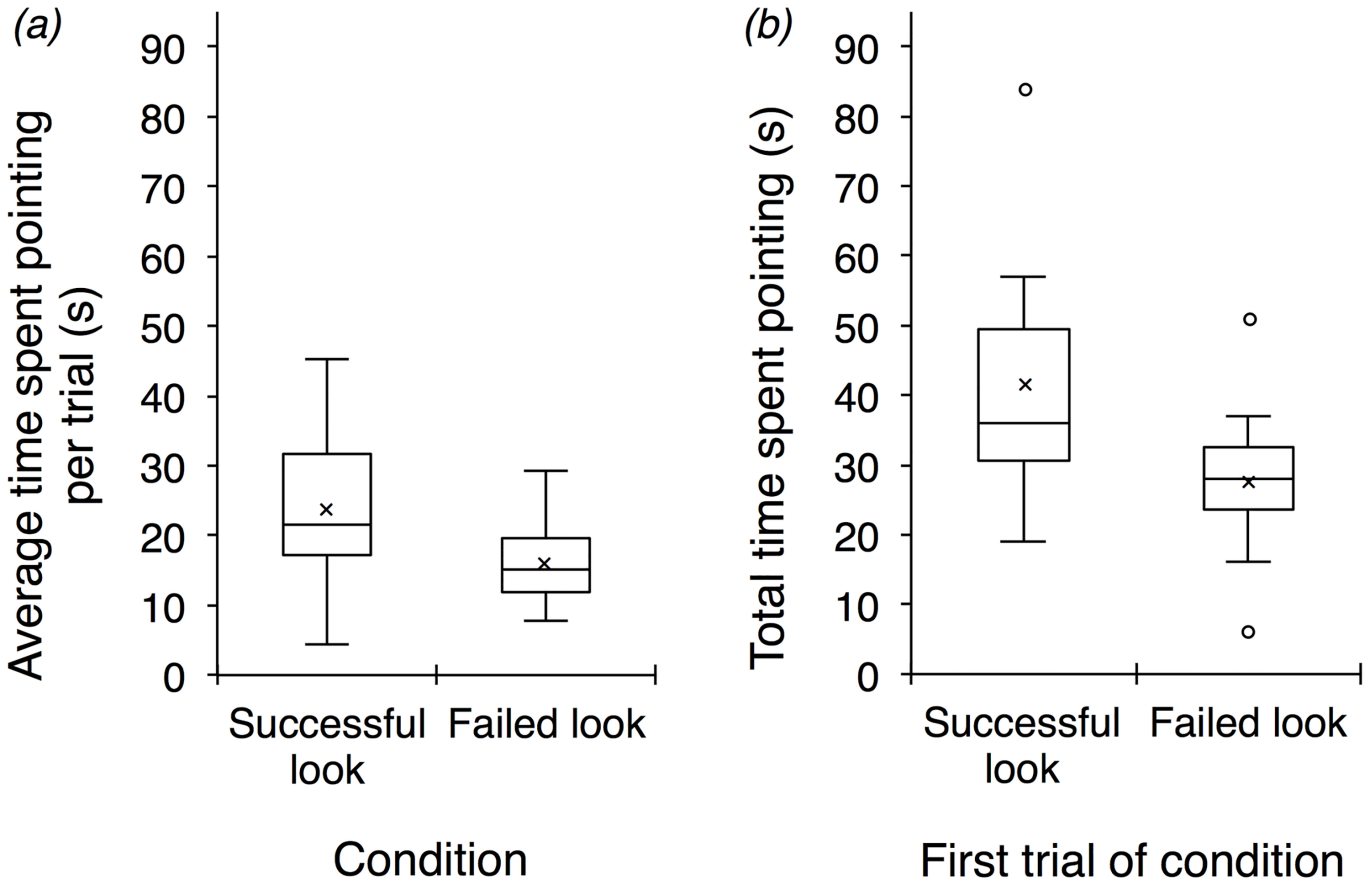
(*a*) On average, subjects spent more time pointing at the grapes per trial in the successful-look condition than in the failed-look condition (*p* = 0.016). (*b*) Subjects also spent more time pointing at the grapes in the first trial of the successful-look condition than in the first trial of the failed-look condition (*p* = 0.009). Results depicted are for points with eye contact. Box plots represent the interquartile range, minimum, and maximum values; depicted also are the means (crosses), medians (horizontal lines), and outliers (circles).

In contrast to our prediction, we found that the average time that subjects spent pointing at the grapes per trial was greater in the successful-look condition than in the failed-look condition. This was the case when considering only those points with eye contact (Wilcoxon signed ranks test: *Z* = -2.35, *p* = 0.016; Fig 2a and Table 4), as well as all pointing behaviors with and without eye contact (Wilcoxon signed rank test: Z = -2.43, *p* = 0.012). Subjects also spent more time pointing at the grapes in the first trial of the successful-look condition than in the first trial of the failed-look condition. This was the case for pointing gestures with eye contact (Wilcoxon signed ranks test: *Z* = -2.49, *p* = 0.009; Fig 2b), as well as all pointing behaviors (Wilcoxon signed rank test: Z = -2.27, *p* = 0.021). Thus, subjects did not spend more time pointing at the grapes when the experimenter failed to look at the indicated object; instead, they spent more time pointing at the grapes when the experimenter successfully looked at the indicated object.

**Table 4:** Individual subject performance*.

Name	Successful-look condition		Failed-look condition	
	Average number of points per trial	Average time spent pointing per trial (s)	Average number of points per trial	Average time spent pointing per trial (s)
Alex	5.00	12.50	5.75	7.75
Alexandra	3.00	18.00	5.25	19.25
Annet	6.25	21.00	6.50	14.75
Fimi	4.25	22.50	2.50	8.50
Jahaga	9.50	45.25	7.25	23.00
Kara	9.00	22.00	5.75	12.50
Kofi	7.25	32.00	10.25	29.25
Limbuko	1.33	4.33	4.00	10.00
Luiza	8.33	31.67	3.75	12.75
Natascha	3.25	15.25	3.00	17.00
Pia	7.25	37.50	4.25	20.25
Sandra	8.00	20.25	7.25	15.50

* Points with eye contact directed at the grapes

We examined the frequency of points across the successful-look and failed-look conditions in order to compare our results with those studies that have measured gestural persistence in terms of frequency (see Methods). We found no difference in the average number of points directed at the grapes across these two conditions. This was the case for points with eye contact (Wilcoxon signed ranks test: Z = -0.78, *p* = 0.435), as well as all pointing behaviors with and without eye contact (Wilcoxon signed ranks test: *Z* = -0.67, *p* = 0.503). We also found no difference in the number of points directed at the grapes during the first trial across these two conditions. This was the case for those points with eye contact (Wilcoxon signed ranks test: Z = -1.12, p = 0.263), as well as all pointing behaviors (Wilcoxon signed ranks test: *Z* = -0.87, *p* = 0.384).

### Time spent away from the experimental window

**Fig 3:**
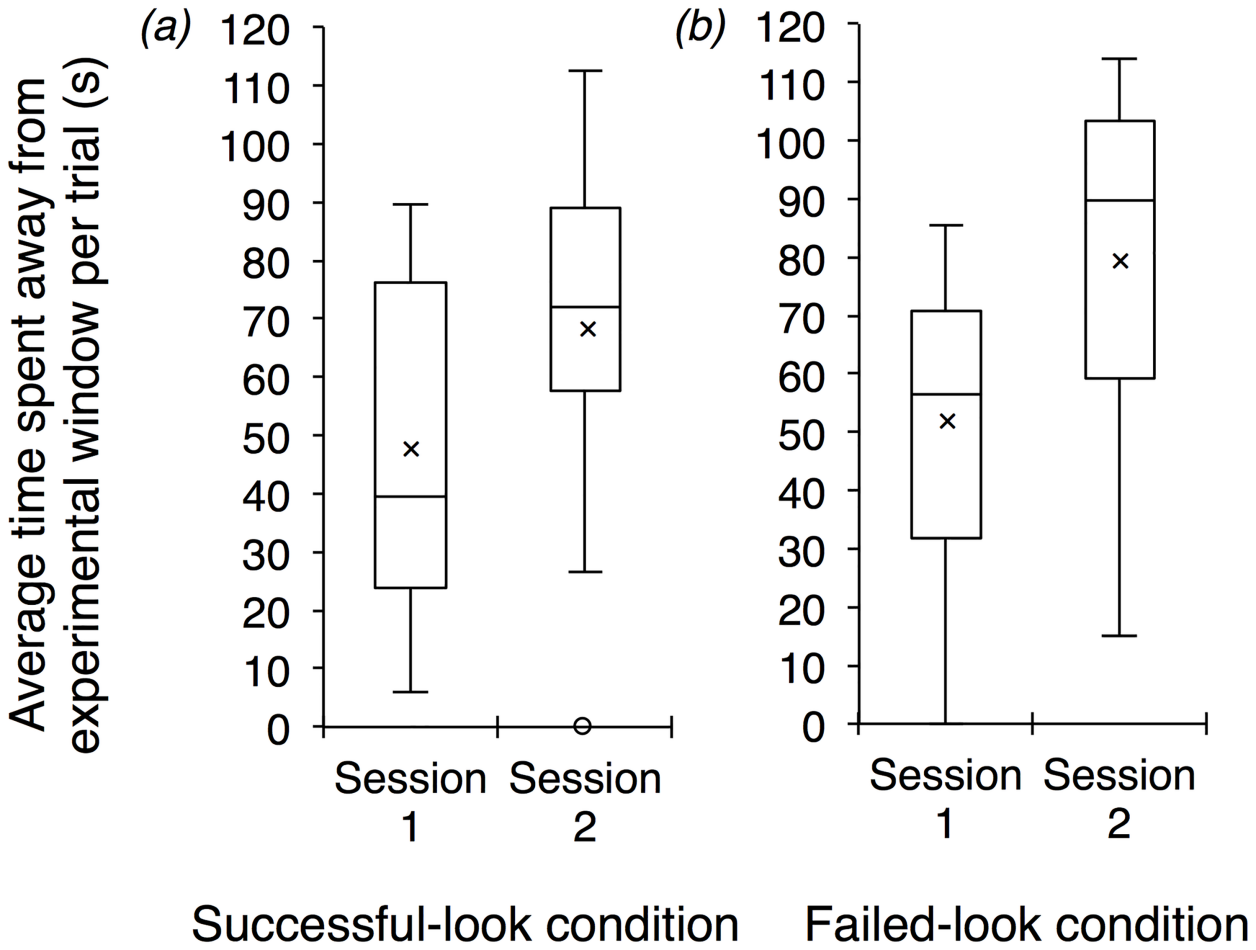
(*a*) We found no significant difference in the average time that subjects spent away from the experimental window per trial between the sessions of the successful-look condition (*p* = 0.176). (*b*) On average, subjects spent more time away from the experimental window per trial during the second session of the failed-look condition compared to the first session (*p* = 0.009). Box plots represent the interquartile range, minimum, and maximum values; depicted also are the means (crosses), medians (horizontal lines), and outliers (circles).

**Fig 4:**
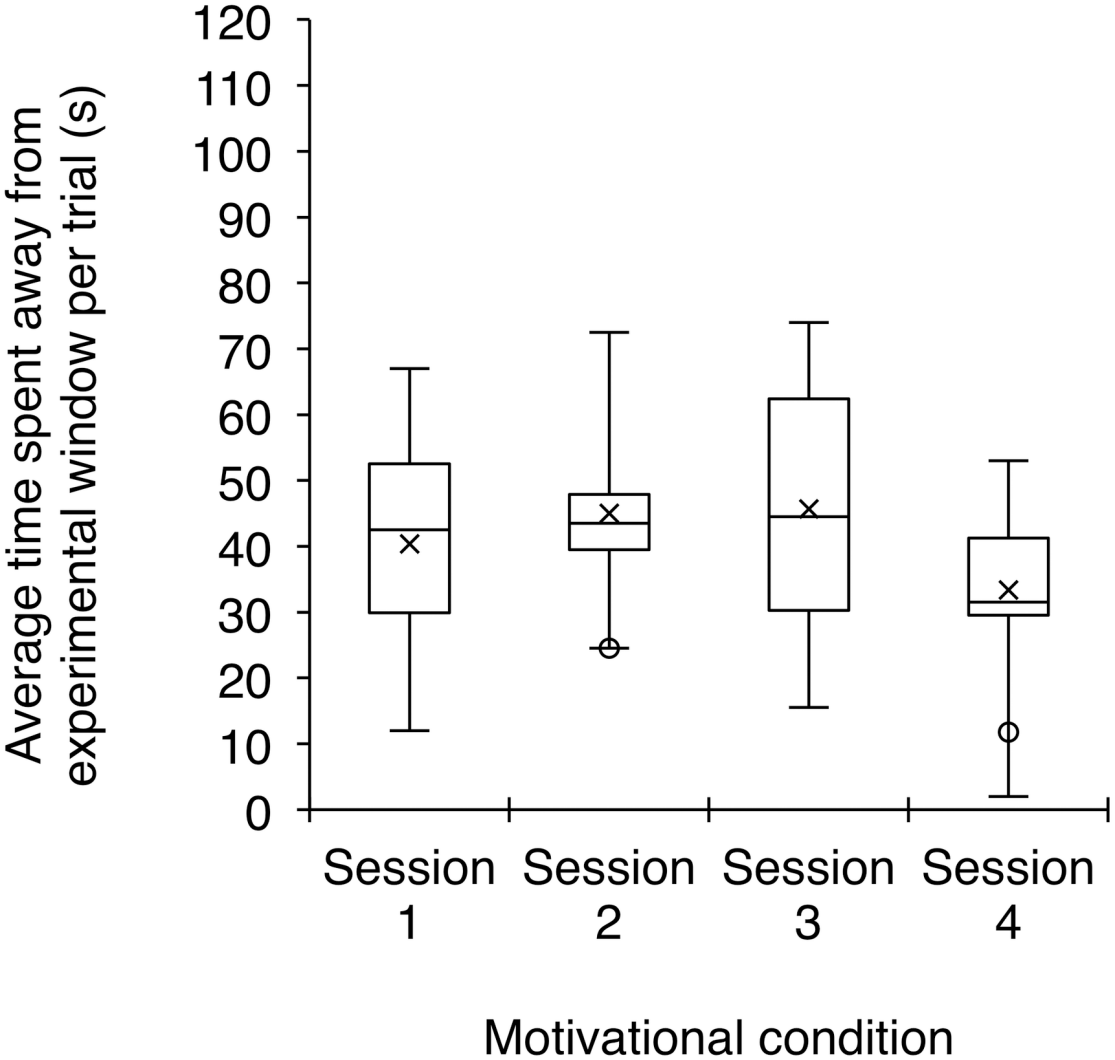
We found no difference in the average time that subjects spent away from the experimental window per trial across the four sessions of the motivational condition. Box plots as described in Fig 3.

In order to monitor the level of participation of subjects in this study, we recorded the duration of time that individuals spent away from the experimental window over the course of each condition. Comparing the two sessions of the successful-look condition revealed no significant difference in the time that subjects spent away from the experimental window (Wilcoxon signed ranks test: *Z* = -1.41, *p* = 0.176; Fig 3a). In contrast, in the failed-look condition, we found that subjects spent more time away from the experimental window in the second session than in the first session (Wilcoxon signed ranks test: *Z* = -2.51, *p* = 0.009; Fig 3b). Across the four sessions of the motivational condition, we found no difference in the time spent away from the experimental window (Friedman Test *χ*^*2*^ = 2.70, *df* = 3, *p* = 0.465; Fig 4).

### Does the percentage of pointing gestures directed at the grapes vary across or within conditions?

**Fig 5:**
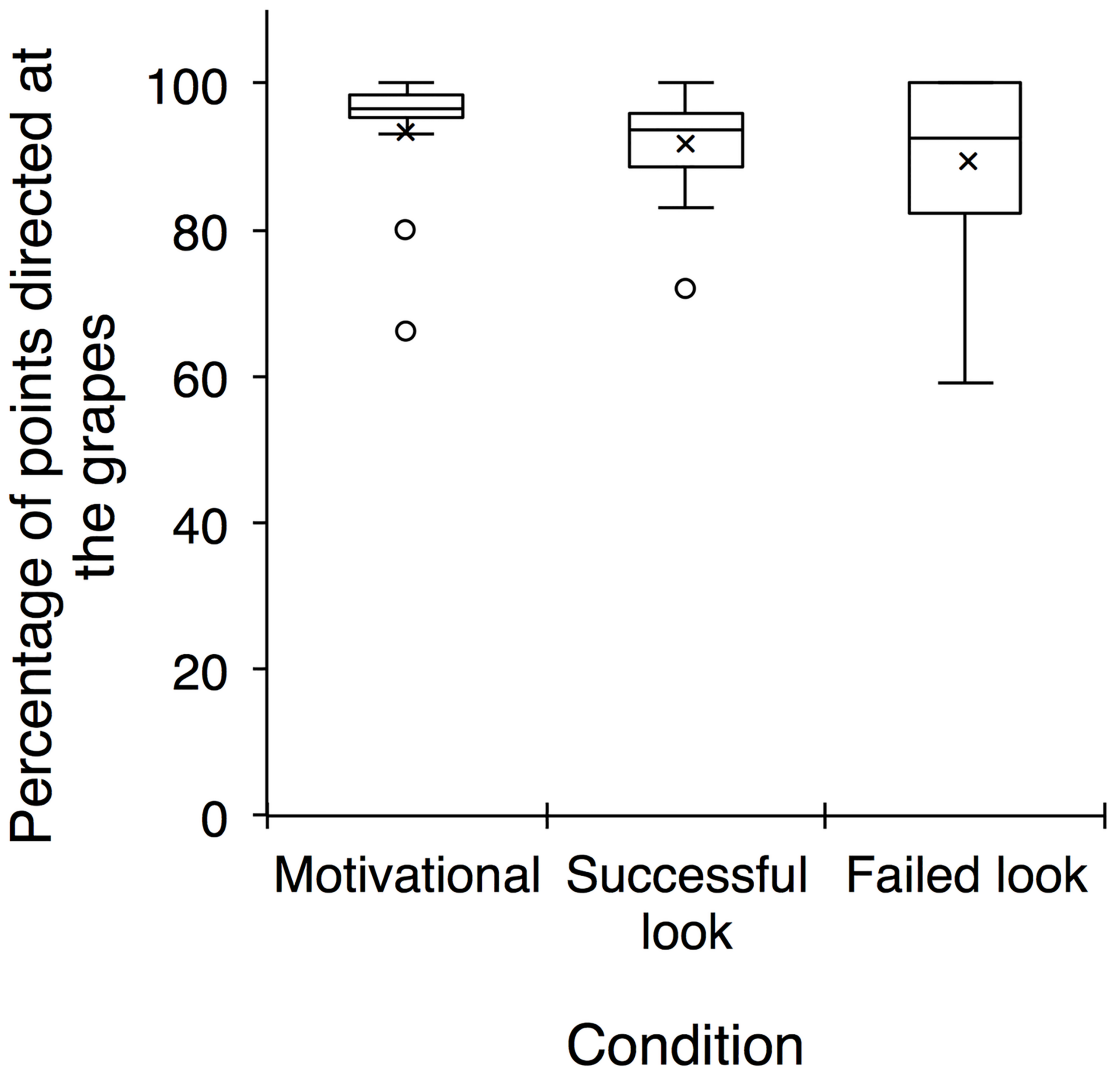
The average percentage of points with eye contact directed at the grapes per trial was high in all three conditions with no significant difference across conditions. Box plots represent the interquartile range, minimum, and maximum values; depicted also are the means (crosses), medians (horizontal lines), and outliers (circles).

We looked at the percentage of pointing gestures directed at the grapes relative to the total number of pointing gestures produced. If subjects were pointing referentially with the aim of getting the experimenter to attend to or act on the grapes, then they should direct the majority of their points with eye contact at the grapes. In all three conditions, the average percentage of pointing gestures directed at the grapes per trial was high (motivational 93.25%, successful-look 91.33%, and failed-look 89.5%) with no significant difference across the three conditions (Friedman test: *χ*^*2*^ = 3.33, *df* = 2, *p* = 0.197; Fig 5). In addition, we found no significant differences in the mean percentage of pointing gestures directed at the grapes across the trials within each condition: motivational (Friedman test: *χ*^*2*^ = 3.54, *df* = 7, *p* = 0.831), successful-look (Friedman test: *χ*^*2*^ = 2.55, *df* = 3, *p* = 0.486), failed-look (Friedman test: *χ*^*2*^ = 4.26, *df* = 3, *p* = 0.243). Overall then, subjects directed most of their pointing gestures at the grapes, and this did not change across or within conditions.

## Discussion

We tested two hypotheses concerning the goal of ape pointing; that in the food context studied here, apes point with the primary goal of directing attention or requesting food. Apes cease gesturing when the goal of their gesture has been met and persist gesturing when the goal of their gesture has not been met [7, 12, 13, 32, 46]. We predicted that, if apes point with the primary aim of directing attention, then subjects would spend more time pointing for a recipient that fails to look at the indicated object than a recipient that looks at the indicated object. Additionally, we expected that if apes point with the primary goal of requesting food, then subjects would exhibit no difference in pointing across the successful-look and failed-look conditions because these conditions differ only in the recipient’s looking behavior. We found that the pointing behavior of subjects did differ between the successful-look and failed-look conditions, suggesting that apes do not point with the sole aim of requesting food in this context. However, contrary to our prediction, subjects spent more time pointing when the recipient responded by looking at the grapes (successful-look) than when the recipient responded by looking elsewhere (failed-look), and they did this from the very first trial. This seems to suggest that apes do not point with the sole aim of directing attention. Thus, we are left with the question, why would subjects point longer for a recipient that has already seen the indicated object than for a recipient that has not yet seen this object?

One possible explanation of the above results is that in the food context studied here apes point with the dual goal of directing attention and requesting food (“please look at that and can you give it to me?”). Under this view, a pointing gesture that succeeds in getting a recipient to look at the indicated object is partially successful; thus, it makes sense to continue pointing because both goals of the gesture have not yet been fulfilled. Moreover, we might expect a signaler in this partially successful situation to be encouraged to point longer because the recipient appears to be on the right track. In contrast, a signaler that is faced with a recipient who shows no signs of producing the right response might be led to abandon his completely ineffective communicative strategy. In this case, we would expect signalers to spend less time pointing in the failed-look condition, which is what we found here.

Several lines of evidence support this dual-goal hypothesis. First, although some chimpanzee gestures (like leaf clipping) have “tight meanings” in the sense of being directed at one goal (such as acquiring sexual attention), many other gestures have “loose meanings” in the sense of being used to obtain more than one goal [13]. It is possible that pointing is a polysemous gesture, where “polysemous” here means having multiple specific functions, like directing attention and requesting food. Alternatively, pointing might be a “general purpose” gesture that has a broad, flexible meaning, rather than multiple specific meanings. As Moore [61] observes, polysemous gestures can be difficult to interpret: how does one interpret a gestures like “hand on” that means both “move closer” and “move away”? Instead such a gesture might be best understood as having the general-purpose meaning of “move!” and the further question of how to move is interpreted from the context in which the gesture is used (is it friendly or antagonistic?). Similarly, ape pointing might have the general meaning of “please act on this object” where “act” can be understood very broadly as anything from look at it, give it to me, or use it (to perform some other desirable action). If this were the case, we might further ask what aspects of the context signalers and recipients rely on to disambiguate the potential meanings of their pointing gestures and whether signalers themselves provide clues on how to correctly interpret their points.

The results of Cartmill and Byrne [7] provide further support for the dual-goal hypothesis (whether understood as polysemous or general purpose). They examined how orangutans responded when their gestural requests for food were met with partial understanding (the recipient of the gesture handed over half of the requested food) versus complete misunderstanding (the recipient handed over a different food item) (see also [36, 46]). They found that subjects tended to repeat gestures that led to partial understanding and avoid gestures that led to complete misunderstanding. As noted above, it is plausible that the subjects in this study were employing a similar strategy—spending more time pointing for the recipient exhibiting partial understanding (one who looks at the food, but does not retrieve it) and less for the recipient exhibiting complete misunderstanding (one who neither looks at the food nor retrieves it), which would suggest that both goals were part of the intended message.

As noted by a reviewer, an assessment of how subjects behaved during the motivational condition might provide additional support for the dual-goal hypothesis. Subjects remained engaged (as measured by the time spent at the experimental window) with the experimenter over the course of both the successful-look and motivational conditions. In contrast, their engagement decreased in the second session of the failed-look condition. Subjects thus appeared motivated to engage the experimenter who either looked at the food or looked at the food and delivered it, suggesting that both of these experimenters were viewed as participants worth engaging. One might object that if subjects were pointing with the dual goal of directing attention and requesting food, then they should cease pointing in the motivational condition because in this condition both of these goals were met. Five grapes were available in the motivational condition, however—obtaining one grape meant that there were still four left to obtain. On the assumption that apes generally prefer more grapes than fewer, the dual-goal hypothesis would predict that apes would only cease pointing when all the grapes had been consumed. A post-hoc analysis of the motivational condition shows that this was the case. In 92% of the motivational trials, subjects did not produce a single pointing behavior after the last of the five grapes was received (S1 Table). (This excludes those points produced while the final grape was being taken down from the grapevine and passed to the subject—that is, within 1–2 seconds of the point that led to that final grape being retrieved.) On only eight trials did three subjects (Limbuko in four trials; Kofi in three trials, and Fimi in one trial) point after the final grape was received. On two occasions Limbuko produced two points in quick succession (less than one second apart); in the other six instances only a single point was produced. Thus, across the entire motivational condition for all subjects (in which 922 points were produced, adding up to 2,764 seconds of pointing), ten points (18 seconds of pointing in total) were produced after all five grapes were gone. On all but one of these occasions, the experimenter handed the empty grapevine to the subject, which the subject took. No points were produced after the grapevine was also gone. Although trials were only 120 seconds long, the fact that most subjects did not produce points after the five grapes were gone cannot be attributed to them running out of time. On average, across all subjects, all five grapes were received 48 seconds before the trial ended with a maximum average time remaining per subject of 75 seconds and a minimum of 33 seconds.

We designed this experiment on the assumption that ape pointing has a relatively tight meaning corresponding to the signaler’s primary goal—either directing attention or requesting food. If apes instead point with multiple goals, then identifying those goals and teasing them apart will require different methods. Future studies might benefit from examining ape pointing in contexts that do not include highly desirable food items in order to better assess the role that directing attention might play as part of the signaler’s goal. Further studies might also benefit from looking at the full range of gestural and vocal strategies employed by apes in pointing contexts, as these communicative strategies might work together to convey the message intended by the signaler. Lastly, more discussion is needed concerning how to operationally define gestural persistence. As noted above, past gestural-persistence studies have focused on gestural frequency or rate [7, 36], while we relied on total pointing durations here (see Methods). Finding the best operational definition of gestural persistence given the unique communicative and experimental context under consideration is important for determining the function of pointing and other primate gestures.

Three final findings of this study are worth highlighting. First, the relationship between pointing and eye contact in apes has been documented in the past [62, 63]; however, few studies have examined eye contact while pointing using video analysis, as we have attempted here. Others have noted that it is difficult to achieve inter-observer reliability on video in great apes [32]. We can confirm this with respect to eye contact, as our agreement was moderate, even though we used three cameras and a well-lit observation room. Previous pointing studies have also focused on gaze alternation: we did not do this here as it would requiring establishing two directions of gaze—eye contact with the experimenter and direct gaze at the food. Although a large majority of the points documented in this study seemed to be made with eye contact (as reported by both the coders and the experimenters interacting live with the subjects), more sophisticated eye tracking techniques are needed to confirm this. Second, previous studies have suggested that apes use pointing gestures referentially [36]. If our observations of frequent eye contact are correct, then this experiment provides additional support for the referential nature of ape pointing, showing that the majority of pointing gestures across all conditions were directed at the grapes. Lastly, prior studies have suggested that gestural communication in chimpanzees is lateralized [62, 63]. Our assessment based on HI values supports this finding: out of the twelve subjects studied here, four exhibited a strong preference for pointing with the right hand and one for pointing with the left hand even though the location of the grapes was counterbalanced across trials.

Over the last decade, it has become clear that great apes are sensitive to the attentional states of others in a wide variety of contexts. This study contributes to this literature by showing that chimpanzees and bonobos are sensitive to the differential looking behavior of agents in response to pointing gestures. This means that communicative adjustments to gaze are not limited to those gestures commonly found in the wild, but extend to signals that depend on the presence of a unique suite of environmental factors for their development [64]. Furthermore, we found no support for the hypotheses that apes point with the sole goal of directing attention or the sole goal of requesting food, and have instead suggested that pointing might be best understood as either a polysemous gesture with multiple specific meanings or a general-purpose gesture with a broad meaning that depends on the context for further specification. If ape pointing were a context-dependent, general-purpose gesture, then this would be an important similarity to how pointing is used and understood by humans from 18 months of age [65, 66].

## Supporting information

S1 Table Pointing gestures across all conditions. The 1,690 pointing gestures recorded across the motivational (M), failed-look (F), and successful-look (S) conditions. Data include point durations (in seconds), hand used (left or right), eye contact (yes or no), point direction (left, right, or center), and grape position (left or right).
(XLSX)

## References

[1] Call J, Tomasello M (eds) (2007) The gestural communication of apes and monkeys. Mahwah: Lawrence Erlbaum Associates

[2] Pollick AS, de Waal FBM (2007) Ape gestures and language evolution. Proc Natl Acad Sci USA 104:8184–8189 doi: 10.1073/pnas.0702624104 17470779 10.1073/pnas.0702624104 PMC1876592

[3] Leavens DA, Hostetter AB, Wesley MJ, Hopkins WD (2004) Tactical use of unimodal and bimodal communication by chimpanzees, *Pan troglodytes*. Anim Behav 67:467–476

[4] Liebal K, Call J, Tomasello M (2004) Use of gesture sequences in chimpanzees (*Pan troglodytes*). Am J Primatol 64:377–396 doi: 10.1002/ajp.20087 15580580 10.1002/ajp.20087

[5] Kaminski J, Call J, Tomasello M (2004) Body orientation and face orientation: two factors controlling apes’ begging behavior from humans. Anim Cogn 7:216–223 doi: 10.1007/s10071-004-0214-2 15034765 10.1007/s10071-004-0214-2

[6] Poss SR, Kuhar C, Stoinski TS, Hopkins WD (2006) Differential use of attentional and visual communicative signaling by orangutans (*Pongo pygmaeus*) and gorillas (*Gorilla gorilla*) in response to the attentional status of a human. Am J Primatol 68:978–992 doi: 10.1002/ajp.20304 16967515 10.1002/ajp.20304 PMC2018749

[7] Cartmill EA, Byrne RW (2007) Orangutans modify their gestural signaling according to their audience‘s comprehension. Curr Biol 17:1345–1348 doi: 10.1016/j.cub.2007.06.069 17683939 10.1016/j.cub.2007.06.069

[8] Hostetter AB, Russell JL, Freeman H, Hopkins WD (2007) Now you see me, now you don’t: evidence that chimpanzees understand the role of the eyes in attention. Anim Cogn 10:55–62 doi: 10.1007/s10071-006-0031-x 16847659 10.1007/s10071-006-0031-x PMC2080772

[9] Tempelmann S, Kaminski J, Liebal K (2011) Focus on the essential: all great apes know when others are being attentive. Anim Cogn 14(3):433–439 doi: 10.1007/s10071-011-0378-5 21258952 10.1007/s10071-011-0378-5

[10] Meunier H, Prieur J, Vauclair J (2013) Olive baboons communicate intentionally by pointing. Anim Cogn 16(2): 155–163. doi: 10.1007/s10071-012-0558-y 22955704 10.1007/s10071-012-0558-y

[11] Hobaiter C, Byrne RW (2011) The gestural repertoire of the wild chimpanzee. Anim Cogn 14:745–767 doi: 10.1007/s10071-011-0409-2 21533821 10.1007/s10071-011-0409-2

[12] Cartmill EA, Byrne RW (2010) Semantics of primate gestures: intentional meanings of orangutan gestures. Anim Cogn 13(6):793–804 doi: 10.1007/s10071-010-0328-7 20563619 10.1007/s10071-010-0328-7

[13] Hobaiter C, Byrne RW (2014) The meanings of chimpanzee gestures. Curr Biol 24:1596–1600 doi: 10.1016/j.cub.2014.05.066 24998524 10.1016/j.cub.2014.05.066

[14] Pika S, Liebal K, Tomasello M (2005) Gestural communication in subadult bonobos (*Pan paniscus*): repertoire and use. Am J Primatol 65:39–61 doi: 10.1002/ajp.20096 15645456 10.1002/ajp.20096

[15] Liebal K, Pika S, Tomasello M (2006) Gestural communication of orangutans (*Pongo pygmaeus*). Gesture 6:1–38

[16] Genty E, Breuer T, Hobaiter C, Byrne R (2009) Gestural communication of the gorilla (*Gorilla gorilla*): repertoire, intentionality and possible origins. Anim Cogn 12:527–546 doi: 10.1007/s10071-009-0213-4 19184669 10.1007/s10071-009-0213-4 PMC2757608

[17] Leavens DA, Russell JL, Hopkins WD (2010) Multimodal communication by captive chimpanzees (*Pan troglodytes*). Anim Cogn 13:33–40 doi: 10.1007/s10071-009-0242-z 19504272 10.1007/s10071-009-0242-z PMC2797826

[18] Tomasello M (2008) Origins of human communication. Cambridge: MIT Press

[19] Hostetter AB, Cantero M, Hopkins WD (2001) Differential use of vocal and gestural communication by chimpanzees (*Pan troglodytes*) in response to the attentional status of a human (*Homo sapiens*). J Comp Psychol 115(4):337–343 doi: 10.1037//0735-7036.115.4.337 11824896 10.1037//0735-7036.115.4.337 PMC2080764

[20] Hopkins WD, Taglialatela JP, Leavens DA (2007) Chimpanzees differentially produce novel vocalizations to capture the attention of a human. Anim Behav 73:281–286 doi: 10.1016/j.anbehav.2006.08.004 17389908 10.1016/j.anbehav.2006.08.004 PMC1832264

[21] Tomasello M, Call J, Nagell K, Olguin R, Carpenter M (1994) The learning and use of gestural signals by young chimpanzees: A trans-generational study. Primates 35(2):137–154

[22] Liebal K, Pika S, Call J, Tomasello M (2004) To move or not to move: how apes adjust to the attentional state of others. Interact Stud 5(2):199–219

[23] Bräuer J, Call J, Tomasello M (2007) Chimpanzees really know what others can see in a competitive situation. Anim Cogn 10:439–448 doi: 10.1007/s10071-007-0088-1 17426993 10.1007/s10071-007-0088-1

[24] Melis A, Call J, Tomasello M (2006) Chimpanzees conceal visual and auditory information from others. J Comp Psychol 120:154–162 doi: 10.1037/0735-7036.120.2.154 16719594 10.1037/0735-7036.120.2.154

[25] Halina M (2015) There is no special problem of mindreading in nonhuman animals. Philos Sci 82(3):473–490

[26] Halina M (2017) What apes know about seeing In: Andrews K, Beck J (eds) Routledge Handbook of Philosophy of Animal Minds. New York: Routledge, pp 238–246,

[27] Lurz R, Krachun C, Mahovetz L, Wilson MJG, Hopkins W (2018) Chimpanzees gesture to humans in mirrors: using reflection to dissociate seeing from line of gaze. Anim Behav 135: 239–249 29610539 10.1016/j.anbehav.2017.11.014 PMC5877477

[28] Hare B, Call J, Agnetta B, Tomasello M (2000) Chimpanzees know what conspecifics do and do not see. Anim Behav 59:771–785 doi: 10.1006/anbe.1999.1377 10792932 10.1006/anbe.1999.1377

[29] Hare B, Call J, Tomasello M (2006) Chimpanzees deceive a human by hiding. Cognition 1010:495–514 10.1016/j.cognition.2005.01.011 16412413

[30] Call J, Tomasello M (2008) Does the chimpanzee have a theory of mind? 30 years later. Trends Cogn Sci 12(5):187–192 doi: 10.1016/j.tics.2008.02.010 18424224 10.1016/j.tics.2008.02.010

[31] Leavens DA, Hopkins WD (1999) The whole-hand point: the structure and function of pointing from a comparative perspective. J Comp Psychol 113(4):417–425 10608565 10.1037/0735-7036.113.4.417 PMC2080771

[32] Leavens DA, Russell JL, Hopkins WD (2005) Intentionality as measured in the persistence and elaboration of communication by chimpanzees (*Pan troglodytes*). Child Dev 76(1):291–306 doi: 10.1111/j.1467-8624.2005.00845.x 15693773 10.1111/j.1467-8624.2005.00845.x PMC2043155

[33] Inoue-Nakamura N, Matsuzawa T (1997) Development of stone tool use by wild chimpanzees (Pan troglodytes). J Comp Psychol 111:159–173 9170281 10.1037/0735-7036.111.2.159

[34] Veà JJ, Sabater-Pi (1998) Spontaneous pointing behavior in the wild pygmy chimpanzee (*Pan paniscus*). Folia Primatol 69:289–290 doi: 10.1159/000021640 9751833 10.1159/000021640

[35] Hobaiter C, Leavens DA, Byrne RW (2014). Deictic gesturing in wild chimpanzees (*Pan troglodytes*)? Some possible cases. J Comp Psychol 128(1): 82–87. doi: 10.1037/a0033757 24040760 10.1037/a0033757

[36] Leavens DA, Hopkins WD, Thomas RK (2004) Referential communication by chimpanzees (*Pan troglodytes*). J Comp Psychol 118(1):48–57 doi: 10.1037/0735-7036.118.1.48 15008672 10.1037/0735-7036.118.1.48

[37] Gómez JC (1990) The emergence of intentional communication as a problem-solving strategy in the gorilla In: Parker ST, Gibson KR (eds) “Language” and Intelligence in Monkeys and Apes. Cambridge: Cambridge University Press, pp 333–355

[38] Call J, Tomasello M (1994) Production and comprehension of referential pointing by orangutans (*Pongo pygmaeus*). J Comp Psychol 108:307–317 7813191 10.1037/0735-7036.108.4.307

[39] Zimmermann F, Zemke F, Call J, Gómez JC (2009) Orangutans (*Pongo pygmaeus*) and bonobos (*Pan paniscus*) point to inform a human about the location of a tool. Anim Cogn 12:347–358 doi: 10.1007/s10071-008-0194-8 18953583 10.1007/s10071-008-0194-8

[40] Bullinger AF, Zimmermann F, Kaminski J, Tomasello M (2010) Differential social motives in the gestural communication of chimpanzees and human children. Dev Sci 14(1): 58–68 10.1111/j.1467-7687.2010.00952.x 21159088

[41] Savage-Rumbaugh S, McDonald K (1988) Deception and social manipulation in symbol-using apes In: Byrne RW, Whiten A (eds) Machiavellian Intelligence: Social expertise and the evolution of intellect in monkeys, apes, and humans. New York: Clarendon Press, pp 224–237

[42] Pika S, Mitani JC (2006) Referential gestural communication in wild chimpanzees (*Pan troglodytes*). Curr Biol 16:R191–R192 doi: 10.1016/j.cub.2006.02.037 16546066 10.1016/j.cub.2006.02.037

[43] Pika S, Mitani JC (2009) The directed scratch: Evidence for a referential gesture in chimpanzees In: Botha R, Knight C (eds) The Prehistory of Language. Oxford: Oxford University Press, pp 166–180

[44] Tomasello M, Gust D, Frost GT (1989) A longitudinal investigation of gestural communication in young chimpanzees. Primates 30(1):35–50

[45] Russell CL, Bard KA, Adamson LB (1997) Social referencing by young chimpanzees (*Pan troglodytes*). J Comp Psychol 111(2):185–193 9170283 10.1037/0735-7036.111.2.185

[46] Roberts AI, Vick SJ, Buchanan-Smith HM (2013) Communicative intentions in wild chimpanzees: persistence and elaboration in gestural signaling. Anim Cogn 16:187–196. doi: 10.1007/s10071-012-0563-1 23053796 10.1007/s10071-012-0563-1

[47] Roberts AI, Vick S, Roberts SGB, Menzel CR (2014) Chimpanzees modify intentional gestures to coordinate a search for hidden food. Nat Commun 5:3088 doi: 10.1038/ncomms4088 24430433 10.1038/ncomms4088 PMC4350813

[48] Liebal K, Call J (2011) The origins of non-human primates’ manual gestures. Philos T R Soc B 367: 118–128 10.1098/rstb.2011.0044 PMC3223783 22106431

[49] Hopkins WD, Cantero M (2003) From hand to mouth in the evolution of language: the influence of vocal behavior on lateralized hand use in manual gestures in chimpanzees (*Pan troglodytes*). Developmental Sci 6: 55–61

[50] Bard KA, Leavens DA (2014) The importance of development for comparative primatology. Annu Rev Anthropol 43:183–200

[51] Tomasello M, Carpenter M, Hobson RP (2005) The emergence of social cognition in three young chimpanzees. Monogr Soc Res Child 28:i–152 10.1111/j.1540-5834.2005.00324.x 16156847

[52] Leavens DA (2004) Manual deixis in apes and humans. Interact Stud 5(3):387–408

[53] McNeill D (1992) Hand and mind: what gestures reveal about thought. Chicago: University of Chicago Press

[54] Kendon A (1980) Gesticulation and speech: two aspects of the process of utterance In: Key MR (ed) The Relationship of Verbal and Nonverbal Communication. Berlin: Walter de Gruyter, pp 207–227

[55] Kendon A (2004) Gesture: visible action as utterance. Cambridge: Cambridge University Press

[56] Tomasello M, George B, Kruger A, Farrar J, Evans E (1985) The development of gestural communication in young chimpanzees. J Hum Evol 144:175–186

[57] Gómez JC (1996) Ostensive behavior in great apes: the role of eye contact In: Russon AE, Bard KA, Parker ST (eds) Reaching into Thought: The Minds of the Great Apes. Cambridge: Cambridge University Press, pp 131–151

[58] Hopkins WD, Bard KA (1993) Hemispheric specialization in infant chimpanzees (*Pan troglodytes*): Evidence for a relation with gender and arousal. Dev. Psychobiol 26:219–235 doi: 10.1002/dev.420260405 8354427 10.1002/dev.420260405

[59] Hopkins WD, Bard KA (1995) Evidence of asymmetries in spontaneous head turning in infant chimpanzees (*Pan troglodytes*). Behav Neurosci 109:808–812 7576226 10.1037//0735-7044.109.4.808 PMC2080770

[60] Hopkins WD (1999) On the other hand: Statistical issues in the assessment and interpretation of hand preference data in nonhuman primates. Int J Primatol 20(6):851–862

[61] Moore R (2014) Ape gestures: interpreting chimpanzee and bonobo minds. Curr Biol 24(24):R645–R647 25050960 10.1016/j.cub.2014.05.072

[62] Leavens DA, Hopkins WD, Bard KA (1996) Indexical and referential pointing in chimpanzees (*Pan troglodytes*). J Comp Psychol 110:346–353 8956506 10.1037/0735-7036.110.4.346 PMC2175394

[63] Leavens DA, Hopkins WD (1998) Intentional communication by chimpanzees: a cross-sectional study of the use of referential gestures. Dev Psychol 34:813–822 9779730 10.1037//0012-1649.34.5.813 PMC2080769

[64] Leavens DA, Hopkins WD, Bard KA (2005) Understanding the point of chimpanzee pointing: epigenesis and ecological validity. Curr Dir Psychol Sci 14(4):185–189 doi: 10.1111/j.0963-7214.2005.00361.x 18159225 10.1111/j.0963-7214.2005.00361.x PMC2151757

[65] Liebal K, Behne T, Carpenter M, and Tomasello M (2009) Infants use shared experience to interpret pointing gestures. Developmental Sci 12(2) 264–271 10.1111/j.1467-7687.2008.00758.x 19143799

[66] Liebal K, Carpenter M, Tomasello M (2010) Infants’ use of shared experience in declarative pointing. Infancy 15(5) 545–556 10.1111/j.1532-7078.2009.00028.x 32693511

